# Serum levels of interleukin-18, CXCL9 and IFN-γ in Still’s disease complicated by macrophage activation syndrome

**DOI:** 10.1093/rap/rkae078

**Published:** 2024-06-11

**Authors:** Inga Z Turtsevich, Kimberly C Gilmour, Ying Hong, Despina Eleftheriou, Paul A Brogan

**Affiliations:** Department of Infection, Immunity and Inflammation, University College London Great Ormond Street Institute of Child Health, London, UK; Department of Paediatric Rheumatology, Great Ormond Street Hospital for Children, NHS Foundation Trust, London, UK; Department of Immunology, Great Ormond Street Hospital for Children, NHS Foundation Trust, London, UK; Department of Infection, Immunity and Inflammation, University College London Great Ormond Street Institute of Child Health, London, UK; Department of Infection, Immunity and Inflammation, University College London Great Ormond Street Institute of Child Health, London, UK; Department of Paediatric Rheumatology, Great Ormond Street Hospital for Children, NHS Foundation Trust, London, UK; Department of Infection, Immunity and Inflammation, University College London Great Ormond Street Institute of Child Health, London, UK; Department of Paediatric Rheumatology, Great Ormond Street Hospital for Children, NHS Foundation Trust, London, UK

Key message

- IL-18 is a promising therapeutic target for Still’s–macrophage activation syndrome (MAS) but cannot differentiate MAS from primary haemophagocytic lymphohistiocytosis.

Dear Editor, Haemophagocytic lymphohistiocytosis (HLH) is a rare and potentially life-threatening syndrome involving hyperactivation of cytotoxic T cells, NK cells and macrophages, leading to cytokine storm, multi-organ failure and death if not promptly recognized and treated [1]. It is classified as primary [familial (pHLH)] and secondary [sHLH, or macrophage activation syndrome (MAS)]. pHLH is usually driven by genetic mutations impairing cytotoxic function, while sHLH is usually triggered by external factors, including infection and/or complicating rheumatic diseases such as Still’s disease [2]. MAS may occur in up to 46% of paediatric Still’s patients and poses a significant diagnostic challenge [2]. Self-perpetuating activation of T cells and macrophages with sustained production of pro-inflammatory cytokines including IL-18 and IFN-γ are key mediators of MAS [1]. IFN-γ has emerged as an important therapeutic target [3]. IFN-γ exerts its biologic activity predominantly in tissues, thus serum levels are overall a poor biomarker of disease activity [4]. Serum CXCL9 level (downstream of IFN-γ) is a more promising surrogate biomarker for IFN-γ-driven systemic inflammation [3]. More recently, IL-18, an IFN-γ-inducing factor, is gaining attention, with higher levels reported in Still’s–MAS compared with pHLH, prompting some authors to suggest its potential to differentiate MAS from pHLH [5] or as a therapeutic target for MAS [6, 7].

Serum cytokine levels might therefore be used to inform a precision therapeutic approach for MAS. However, data regarding serum levels of these cytokines in health or disease are extremely limited. The primary aim of this study was to document serum levels of IL-18, CXCL9 and IFN-γ in patients with active Still’s–MAS, inactive Still’s–MAS, healthy controls and disease controls (pHLH, or other CTDs without MAS). Second, the potential diagnostic utility of these cytokines to differentiate Still’s–MAS from pHLH was explored.

This study analysed 72 banked serum samples collected between 1 June 2019 and 30 September 2019 (i.e. pre-pandemic) and stored at the Camelia Botnar Immunology Laboratory at Great Ormond Street Hospital (ethics 06/Q0508/16, R&D 05MI12). Disease classification was based on referring physician diagnoses and clinical records, including genetics. The samples included 13 with active Still’s–MAS, 3 with inactive Still’s–MAS, 18 with pHLH [*n* = 7 bi-allelic *PRF1* mutation, *n* = 6 bi-allelic *UNC13D* mutation, *n* = 3 XIAP deficiency, *n* = 1 Griscelli syndrome (bi-allelic *RAB27A* mutation)] and 1 Hermansky–Pudlak syndrome (bi-allelic *HPS3* mutation). Other CTDs without MAS (*n* = 15) were juvenile SLE (*n* = 5), juvenile dermatomyositis (*n* = 5) and juvenile scleroderma (*n* = 5) and 23 non-matched healthy controls.

Serum samples were analysed using the Multiplex immunoassay (Meso Scale Diagnostics, Rockville, MD, USA) for IFN-γ, CXCL9 and IL-18. Standard concentration curves (pg/mL) for each cytokine were determined according to the manufacturer’s recommendations. Statistical analyses employed GraphPad Prism 10 (GraphPad Software, San Diego, CA, USA). Continuous variables were presented as median (range). Group comparisons were assessed using the non-parametric Mann–Whitney U test for two samples and Kruskal–Wallis test for multiple independent samples. Receiver operating characteristics (ROC) curves and the Youden (J) index were used to gauge diagnostic utility of IL-18 for identification of Still’s–MAS *vs* pHLH. All comparisons were based on disease classification, with no age-related subgroup analyses. Significance was set at *P* < 0.05.

IL-18 levels were highest in active Still’s–MAS, followed by pHLH and inactive Still’s–MAS patients (*P* < 0.0001). IL-18 was higher in active Still’s–MAS *vs* pHLH, although this result was not significant (*P* = 0.06) (Table 1). IL-18 was higher in active Still’s–MAS *vs* inactive Still’s–MAS (*P* = 0.014). Of note, however, patients with inactive MAS still had numerically higher levels of all three cytokines compared with healthy controls (patients were too few to perform meaningful statistical analyses). The CXCL9 level was overall higher in MAS and pHLH *vs* healthy controls and CTDs without MAS (*P* < 0.0001) (Table 1). There was no statistical difference in CXCL9 between patients with MAS and pHLH (*P* = 0.77) (Table 1). IFN-γ concentrations did not significantly differ among groups (*P* = 0.122). ROC analyses were performed to explore the diagnostic utility of IL-18 to differentiate active Still’s–MAS from pHLH [5, 6]: at an optimal cut-off level of 30 764 pg/mL, IL-18 demonstrated only modest diagnostic utility (sensitivity 0.615, specificity 0.778, area under the curve 0.705, J index 0.393, *P* = 0.055).

**Table 1.** Serum IL-18, CXCL9 and IFN-γ levels in healthy controls, Still’s–MAS, pHLH and other CTDs

Serum cytokine (pg/mL), median (range)	Healthy controls (*n* = 23)	Active Still’s–MAS (*n* = 13)	Inactive Still’s–MAS (*n* = 3)	pHLH (*n* = 18)	CTDs without MAS (*n* = 15)
IL-18	325 (0.3–734)	38 322 (5872–168 952)	1179 (757–5007)	7306 (2638–33 050)	509 (248–646)
CXCL9	268 (5–924)	5790 (2768–14 415)	7461 (4603–12 470)	5011 (1772–40 521)	674 (243–1136)
IFN-γ	18 (13–26)	33 (18–81)	54 (8–66)	45 (10–743)	16 (12–22)

In conclusion, serum cytokines might in the future inform therapeutic stratification for Still’s–MAS since CXCL9 was already suggested as a biomarker to stratify patients towards IFN-γ-blockade [3]; Shimizu *et al.* found that tocilizumab-treated systemic JIA patients with high IL-18 levels (>47 750 pg/mL) were prone to develop MAS, suggesting IL-1 blockade may be preferred for these cases [7]; and patients with inactive MAS but persistently high IL-18 may be prone to develop Still’s-associated lung disease [8]. Lastly, IL-18 may ultimately prove to be an important direct therapeutic target for Still’s–MAS [8], and a trial in children and adults is now warranted. We suggest, however, that IL-18 levels cannot be used reliably to differentiate MAS from pHLH.

## Data Availability

All data are available upon request.
